# Inferring neural information flow from spiking data

**DOI:** 10.1016/j.csbj.2020.09.007

**Published:** 2020-09-20

**Authors:** Adrià Tauste Campo

**Affiliations:** Centre for Brain and Cognition, Universitat Pompeu Fabra, Ramon Trias Fargas 25, 08018 Barcelona, Spain

**Keywords:** Single neuron, Spike train, Simultaneous recordings, Granger causality, Information flow

## Abstract

The brain can be regarded as an information processing system in which neurons store and propagate information about external stimuli and internal processes. Therefore, estimating interactions between neural activity at the cellular scale has significant implications in understanding how neuronal circuits encode and communicate information across brain areas to generate behavior. While the number of simultaneously recorded neurons is growing exponentially, current methods relying only on pairwise statistical dependencies still suffer from a number of conceptual and technical challenges that preclude experimental breakthroughs describing neural information flows. In this review, we examine the evolution of the field over the years, starting from descriptive statistics to model-based and model-free approaches. Then, we discuss in detail the Granger Causality framework, which includes many popular state-of-the-art methods and we highlight some of its limitations from a conceptual and practical estimation perspective. Finally, we discuss directions for future research, including the development of theoretical information flow models and the use of dimensionality reduction techniques to extract relevant interactions from large-scale recording datasets.

## 1 Introduction

**Fig. 1:**
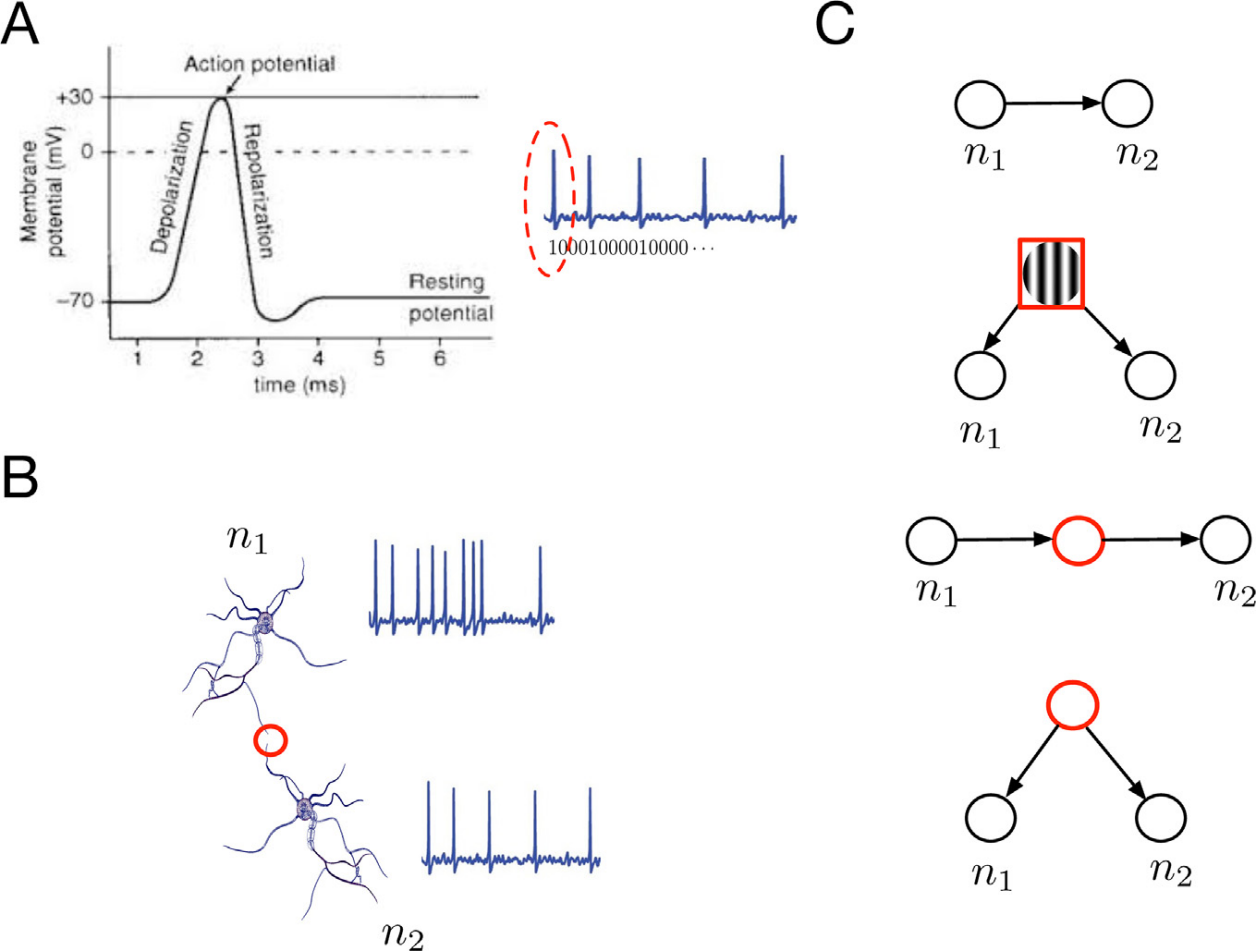
Inferring single-neuron interactions from spiking data. (A) On the left caption, the time course of an action potential (or spike) showing the rise (‘Deplorarization”) and fall (“Repolarization”) of the membrane potential with respect to a background level (“Resting potential”). On the right caption, a depiction of a spike train where the first action potential is highlighted in red. Below the spike train, its usual modelization as a binary sequence of 0s and 1-s (spikes) is correspondingly displayed. (B) A schematic depiction of two neurons, $n_{1}$ and $n_{2}$, with their respective spike trains, displaying a synaptic connection (in red) between $n_{1}$ axon’s terminal and $n_{2}$’s dendrites. (C) Four model configurations that can explain an estimated pairwise statistical dependence between $n_{1}$ and $n_{2}$. On the top caption, a model showing both neurons that are directly connected by a synapse. On the middle-top caption, a model showing a visual stimulus (highlighted in red) exerting a simultaneous effect on both neurons. On the middle-bottom and bottom captions, both neurons being mediated by a third neuron (highlighted in red). The three later examples can be referred to as $n_{1}$ and $n_{2}$ being independent conditioned to or, equivalently, d-separated by either a stimulus (middle-top) or other neurons’ activity (middle-bottom and bottom) [122]. (For interpretation of the references to colour in this figure legend, the reader is referred to the web version of this article.)

A central question in neuroscience research is how the interaction of multiple neurons in the central nervous system leads to cognition. Over years, biology has provided a detailed description of how neurons interact via synapses in terms of electro-chemical processes [1]. This interaction is mainly produced by the propagation of action potentials. An action potential (commonly known as a spike) is generated by the abrupt increase and fall of a neuron’s membrane potential. This change of polarization usually occurs in the soma of the neuron and travels down the neuron’s axon towards its terminal to produce electro-chemical signals that are transmitted to the dendrites of synaptically connected neurons, which in turn generate new action potentials (see Fig. 1A and B). Spike propagation is the main means of cell-to-cell communication in the nervous system. Consequently, spikes are analyzed as the main unit of information conveyed by neurons while their temporal sequence of occurrences, known as a spike train, is conceived as the stream of information that travels through the nerves [2]. The usual mathematical symbolization of spike trains is via a binary sequence of 0s and 1s, where the neuron’s time-binned activity is mapped to 1 for spike occurrences and to 0, otherwise 1 (Fig. 1A). In practice, spikes are measured via extracellular recordings. This type of recordings captures the electrical field generated by the difference in potential between two locations in the extracellular medium [3]. In particular, when these recordings are performed at a very fine scale, spike trains from different neurons can be discriminated by sequentially applying high-frequency filtering, spike detection and spike-sorting algorithms on the recorded signals [4].

Nowadays, current technological advances in neural recording systems have allowed to record the electrical activity of an ever-growing number of simultaneous neurons across many species including humans [5]. With these data, one can formulate the general question: Given a subset of simultaneous spike train recordings from different brain areas, how can we reconstruct to a certain precision interactions between the observed neurons to uncover functionally relevant information flows? Despite the interest on the topic, computational approaches to this question are still limited. Indeed, they are diverse in nature, suffer from technical and conceptual shortcomings and can lead to ambiguous biological interpretations. In this paper, we review the main contributions to the topic as well as discuss new promising directions for further development.

## 2 From cross-correlations to model-based approaches

Since the early birth of simultaneous single-neuron recordings in the mid 1960s [6], neurophysiologists have attempted to jointly analyze and interpret spike trains to provide experimental information about synaptic connections and other potential sources of functional interaction among the detected neurons [7], [8], [9]. The initial tools were based on descriptive pairwise statistics such as cross-correlations between neuron’s spike counts, namely the number of spikes over the entire spike train, [7] and bivariate histograms of spike times [8], which were both computed across experimental repetitions (commonly known as *trials*) from the same pair of neurons. Yet, already in 1967, Perkel et al. admitted some of the principal limitations of interpreting neural interactions via cross-correlation, which also apply to a large ensemble of methods used today [7]. The first limitation is that a pairwise correlation in a neuron pair can be equally explained by a synaptic connection, a third-neuron mediation or by a shared input like stimulus information [7] (see Fig. 1C). The second limitation is that the sequence of trials used for cross-correlation and histogram estimation cannot be in general assumed independent and identically distributed [10] and hence, estimation from multiple trials needs to be performed cautiously. This assumption might be questionable when trials from different days are pooled together or when external and uncontrolled variables (e.g., level of arousal, motivation) have a time-varying effect on subject’s behavioral variables (e.g., task performance) across trials.

By the of the 20th century, several works started to address the above-mentioned concerns. On one hand, the authors in [10] were able to develop a robust method to isolate the residual component of the cross-correlation that only accounted for the effect of the stimulus on each neuron’s activity. On the other, it was showed that cross-correlations could confound distinct sources of potential covariation that included genuine time synchrony as well as externally-driven covariations of independent neural responses [11]. In this situation, heuristic rules [11] and quantitative methods [12] were proposed to help resolve potential ambiguities and improve the interpretation of experimental outcomes.

While some cross-correlation limitations may be tackled by ad hoc methods [10], [12], [13], a more general framework is necessary to simultaneously account for all the confounding sources of covariability [14]. In this context, by the end of the last century, several works started to regard spike train sequences in the frequency domain and made used of Fourier methods and spectral measures of association (e.g. coherence) to characterize distinct sources of influence in single-neuron interactions [15], [16], [17]. For instance, this approach led to identifying common inputs in pairwise interactions [18] and to the development of partial directed coherence [19], [20], a measure of interaction that incorporates directionality and controls for the effect of other observed neurons. In parallel, in the early 2000s, statistical models emerged as a powerful tool to model the influence of covariates such as the stimulus, and the own or other neurons’ previous spiking history [21]. Specifically, model-based approaches are grounded on minimal generative assumptions, i.e., how the observed variables are generated, and typically fit the model parameters using maximum-likelihood estimation [22], i.e., choosing those values that maximize the conditional probability of the observed variables given the parameters. A well-known example in neuroscience is the generalized linear model (GLM), which statistically describes spike trains as an inhomogeneous Poisson point processes whose time-varying intensity (also known as Poisson rate) results from a non-linear function of filters, each processing a different variable influencing the neuron’s activity, such as the stimulus, the spike train own past activity and the spike trains from other neurons [21]. The use of GLMs has been widely applied to study neural interactions in a number of simultaneous studies [23], [24], [25], [26]. For instance, it was showed how retinal cell interactions were more prominent between neighboring cells and how these interactions improved the decoding of visual stimuli [23].

The GLM detailed description of the neurons’ spiking activity comes at the expense of a potentially large number of model parameters. This usually can produce poor performance in generalising the results across different experimental sessions. Several studies have overcome this issue by introducing prior knowledge about the observed data. This includes invoking analytical assumptions on the coupling time-varying functions [23] or modelling interaction sparsity using Bayesian inference [27]. Yet, the application of Bayesian inference in this context has its own limitations. Indeed, modelling neural interactions as Bayesian networks without adequate constraints [28] may be computationally unfeasible. Another critical issue is the fact that GLM assumes parameter invariance during repeated experimental trials as we will discuss in the next section.

## 3 The Granger causality framework: main concept and model-free generalizations

In most applications, a GLM assumes that the underlying Poisson process is stationary within and across trials [21]. Hence, it fits a single coupling filter for each neuron pair across repeated experimental trials, which might obscure the functional relevance of trial-dependent interactions. In particular, trial-to-trial fluctuations can occasionally alter the number of spikes of some driver neurons, producing a larger effect on target neurons during specific trials [29]. We will devote this section to a framework that allows to infer single-trial causal dependencies. We will first state its main concept, we will then define and discuss its generalized information-theoretic formulas and we will conclude by reviewing some applications in neuroscience.

### 3.1 Main concept

In order to analyze single-trial dependencies, an established approach is to model spike trains as binary time series^2^ and resort to the Granger causality^3^ (GC) framework [30], [31]. Granger’s causality is a concept that originated in econometrics in the 1950s whose core idea is the following: a time series X causes^4^ Y if X contains information that helps predict the future of Y better than any information already present in the past of Y, and, if available, in the past of other observed variables Z [31].

### 3.2 Model-free generalizations: directed information

In its original form, GC was conceived to be applied to multivariate linear autoregressive Gaussian models (MVAR) in both temporal [31] and frequency domains [33], [34] but the basic idea can be generalized to arbitrary join probability distributions governing the observed variables. When an estimation method uniquely relies on the joint probability distribution of the observed variables, it is usually referred to as “model-free” in the neuroscience literature as opposed to “model-based” approaches relying on a predefined statistical model. Crucially, because spike trains are naturally modeled as Poisson and not Gaussian processes [21], model-free methods are more suitably than the GC-MVAR to capture the specificities of spiking activity. In fact, a model-free generalization of the GC concept can be found in the information-theoretic concept of directed information. The directed information (DI) is a functional that was originally developed in [35], [36], [37] to study the maximum achievable transmission rates in communication channels with feedback but can also be used to measure causal statistical dependencies between sequences of random variables. Formally, the DI can be defined as the sum of conditional mutual information terms [38], which makes it applicable to arbitrary statistical models and to both discrete and continuous variables.

In the following, we will provide the mathematical definition of DI. Let X,YandZ be three arbitrary variables. The conditional mutual information between *X* and *Y* conditioned on *Z* is defined as

(1) $$I(X\text{;}Y|Z)=E_{\mathrm{XYZ}}\left[\log\frac{P_{Y|X,Z}}{P_{Y|Z}}\right],$$

where $E_{\mathrm{XYZ}}$ denotes the expectation over the joint probability distribution $P_{\mathrm{XYZ}}$. Let us now be more specific and assume that the arbitrary variables above are sequences of random variables. In particular, let us consider two T-length sequences $X^{T}$ and $Y^{T}$ defined as $X^{T}=(X_{1},\ldots ,Y_{T})$ and $Y^{T}=(Y_{1},\ldots ,Y_{T})$. To introduce the DI, we will make use of the mutual information formula for sequences of variables [38]. The mutual information between $X^{T}$ and $Y^{T}$ can be decomposed via the chain rule in the sum of T conditional mutual information terms:

(2) $$I(X^{T}\text{;}Y^{T})=\overset{T}{\underset{t=1}{\sum }}I(Y_{t}\text{;}X^{T}|Y^{t-1}),$$

where the notation $A_{\tau }^{\tau^{\prime }}$ stands for the sequence $A_{\tau }^{\tau^{\prime }}=(A_{\tau },\ldots ,A_{\tau^{\prime }})$, τ⩾τ, for which the subscript is dropped when τ=1 [38]. In contrast to (2), the DI between $X^{T}$ and $Y^{T}$ is defined as

(3) $$I(X^{T}\to Y^{T})=\overset{T}{\underset{t=1}{\sum }}I(Y_{t}\text{;}X^{t}|Y^{t-1}),$$

where, in each summand of (3), the $X^{T}$ appearing in the second argument of (2) has been replaced by $X^{t}$, thus accounting only for the dependency of each $Y_{t}$ on up to the tth element of $X^{T}$. While the mutual information is symmetric, the DI is not, and hence the later yields in general a different value when computed in reverse direction, i.e., from $Y^{T}$ to $X^{T},I(Y^{T}\to X^{T})\;\neq \;I(X^{T}\to Y^{T})$. Although (3) holds for general statistical models, under certain conditions of stationarity and ergodicity it is more convenient to recall its temporal normalized version, known as the DI rate:

(4) $$\frac{1}{T}I(X^{T}\to Y^{T})\text{.}$$

In addition to causal inference, the DI has an operational meaning in different information-theoretic and statistical domains ranging from data compression to channel coding or hypothesis testing [39]. Importantly, the DI is the fundamental limit of communication (that is, the maximum achievable transmission rate) over a certain type of noisy channels when noiseless feedback is present at the transmitter [40], [41]. Hence, the DI is not only a convenient measure of causal dependence between data sequences but it is also the theoretical *answer* to problems involving communication models.

Over the last decade, a number of consistent DI rate estimators have appeared in the literature [29], [42], [43]. For instance, in [29] the authors defined an estimator to infer causal relationships in neural spike trains by assuming a Poisson statistical model and fitting its parameters with GLM over long single trials. Then, the required conditional probabilities of $X^{T}$ and $Y^{T}$ were obtained from the model to be plugged into the DI rate formula (4). In the most general case, however, no information about the underlying model is presumed and the joint probability distribution of $X^{T}$ and $Y^{T}$ needs to be estimated in a non-parametric form. Under this condition, novel DI rate estimators were defined in [42], where the estimator relied on a sequential and universal probability estimation algorithm named context tree weighting (CTW, [44]), and in [43], where the authors analyzed the performance limits of the probability maximum-likelihood estimator. Importantly, in all the above cases, the estimation procedure becomes computationally feasible when the sequences $X^{T}$ and $Y^{T}$ are assumed to be generated according to jointly stationary and ergodic Markov processes [45].

### 3.3 Model-free generalizations: transfer entropy

Consistent with the GC concept, another information-theoretic functional was independently proposed under the name of transfer entropy (TE), aimed to measure causal dependencies between random processes in dynamical systems [46]. Unlike the DI, the TE only applies to pairs of stationary processes, $X_{t}\;\;\text{and}\;\;Y_{t}$, that jointly satisfy the Markov property, i.e.,

(5) $$P_{Y_{t+1}|X^{t},Y^{t}}=P_{Y_{t+1}|X_{t-K+1}^{t},Y_{t-J+1}^{t}}$$

for any t⩾max(J,K), where JandK are the order of each process, respectively. Given (5), the TE between processes $X_{t}\;\;\text{and}\;\;Y_{t}$ is defined as

(6) $$\mathrm{TE}(X\to Y)=I(Y_{t+1}\text{;}X_{t-K+1}^{t}|Y_{t-J+1}^{t}),$$

for any t⩾max(J,K) [47]. Under the usual assumptions of DI rate estimators (stationarity, ergodicity and the Markov property), it can be easily checked that the DI rate converges to the TE in the limit of the sequence length *T* [48]. Furthermore, when these conditions hold in Gaussian models, it can be shown that both DI and TE coincide with the MVAR version of GC [49]. Similarly to MVAR models, the DI, the TE and other GC-derived measures can also be extended via conditioning to measure conditional causal dependencies in multivariate models. Examples of such multivariate extensions have been theoretically proposed for the DI [50], [51], for the TE [52], [53] as well as for other GC-derived measures [54].

### 3.4 Estimation remarks

When estimating model-free GC-derived measures, both the outer expectations and the inner (conditional) probability distributions appearing in (1) are approximated by leveraging a sufficiently large number of temporal samples from the observed time series. Therefore, in this type of estimations, there is a trade-off between the assumptions of stationarity and ergodicity usually holding at short segment lengths and the estimation power requiring lengthy time series. These constraints do not apply in other reviewed methods such as cross-correlation in which samples are obtained from the number of trials over which a certain quantity is averaged. Critically, in neuroscience studies, one may argue that the use of temporal samples (instead of trials) may compromise the inference of the exact times at which spike-train interactions occur. However, over the last years, a few works have shown that interaction times can also be revealed in this framework via delayed versions of the original measures [55] and ad hoc statistical tests (e.g., see Supplementary information in [56]). Finally, the statistical power of model-free GC-derived measures can be assessed by performing nonparametric significance testing of the estimated quantities using methods such as permutation tests [57].

### 3.5 Application to neuroscience studies

Since the early 2000s, a number of data-driven methods derived within the GC framework have been applied to pairs of simultaneously recorded neurons in order to investigate how information flows between brain areas are associated to cognitive functions.

Because GC was originally aimed to analyze continuous-value time series, the classic MVAR formulation of GC [58] is not a priori suitable to deal with binary spike trains. However, some works circumvented this issue by developing variants of the original method. For instance, in experimental studies about visual information processing [59], [60], a non-parametric version of the original GC estimation in the frequency domain [61] was applied to spike trains, thus bypassing the point-process modelling [21]. This approach was specifically tested with recordings from visual areas, while monkeys were exposed to visual stimuli [59], [60]. In this application, Hirabayashi et al. highlighted the temporal recurrence of feedforward and feedback interactions in the same pair of neurons during stimulus presentation [59]. An alternative approach was due to Kim et al. who kept the point process modelling of spike trains [21] and proposed a Poisson-log likelihood version of the original GC measure [58].

The application of the DI to simultaneous single-neuron datasets became specially popular after its adequacy to handle spiking data was demonstrated in [29]. Specifically, in [29], the proposed DI rate estimator was applied to recordings from the primary motor cortex (M1) of a monkey while it performed arm movement tasks according to visual targets. The outcomes of the analysis supported the existence of electrical propagation waves above 10 Hz, which are known to encode information about visual targets in reaching tasks [62]. In addition, a variant of the DI rate estimator introduced in [29] was proposed in [63], which showed an accurate estimation of the conduction delays between neurons in different brain areas during motor tasks performed by rodents and nonhuman primates. On the other hand, time-delayed versions of the CTW-based estimator were elaborated in [56], [64] to infer task-driven directional interactions between the thalamus and the somatosensory area 1 (S1) in monkeys performing a tactile detection task [56], and across cortical somatosensory, premotor and motor areas in monkeys performing a tactile dissemination task [64]. Finally, an extension of the CTW algorithm for non-necessarily finite-order Markov processes [65] was used to estimate the DI rate between neural spike trains from the buccal ganglion of *Aplysia* [66].

## 4 GC limitations: estimation and interpretation

Over the last couple of decades, the GC framework has become one of the main statistical method in neuroscience to analyze neural interactions from a variety of recording modalities including spike trains. Despite its growing popularity, its practical application has also raised some concerns [67], [68], [69], about the computational reliability of the estimated outcomes and their biological interpretation. In this section, we will review two sources of criticism about GC-derived measures: those concerning their estimation, and those related to the information flow interpretation of their outcomes.

### 4.1 Estimation challenges

The original formulation of the GC concept relying on linear Gaussian statistics has been refined in the frequency domain to resolve some of its initial technical limitations such as the bias and high variance of the interactions estimates [61], [58]. However, some additional challenges prevail such as the validity of the linearity and stationarity assumptions, or the effect of temporal sampling [70], [69], [71], which may impair its application to spike train data. In fact, the use of model-free generalizations like the DI or TE resolves the linearity assumption, but it is still susceptible to problems such as the estimation bias or the lack of stationarity in data recordings. Nevertheless, recent works have showed promise in dealing with these later issues. For instance, Schamberg et al. showed that the above reviewed DI estimators are biased when the Markov order of the receiving process $Y^{T}$ is different from the order of the joint processes $\left(X^{T},Y^{T}\right)$ [45]. In addition, they outlined sufficient conditions under which the equal-order Markovian assumption is met and provided a bound for the estimation bias in those cases when such conditions may not be satisfied. To address the non-stationarity problem, Sheikhattar et al. developed a window-based adaptive model that makes uses of point-process modelling and leverages the sparsity of spiking data [72]. They applied this technique to simultaneous recordings from ferrets to describe time-varying top-down and bottom-up interactions between primary auditory area (A1) and prefrontal cortex (PFC) during a tone detection task.

### 4.2 Interpretation issues

One of the fundamental criticism about the GC statistical framework in general, and about GC-derived measures in particular, is the interpretation of the inference outcomes as characterizing information flows between neurons. Importantly, a review of the recent literature [67], [69], [73], [71] readily reveals that some of the controversy mainly arises due to the different notions of information flow that researchers adopt in their studies. Hence, we might start asking the conceptual question: what do we understand by information flow?

To begin with, if measuring information flow means detecting the exchange of information between neuron *A* and neuron *B* through their synaptic connections, then the GC framework (and also the GLM) alone is in general insufficient to address this question. This is because the GC concept and its information-theoretic generalizations are aimed to infer statistical dependencies between observed variables and, therefore, its application to spike train data characterizes single-neuron interactions only at a phenomenological level. As such, GC-derived measures are susceptible to latent confounding effects arising from limited spatial sampling such as the influence of unobserved neurons. Indeed, given the thousands of neurons that may have an effect on a single postsynaptic neuron, the GC estimates are in general not able to discriminate between anatomically direct or indirect connections. Instead, if we wish to make detailed inferences about synaptic connections or other sources of interactions, mechanistic approaches are required. An example of such approaches is dynamic causal modeling (DCM) [74], a widely established framework to analzye coarser neural data modalities like functional magnetic resonance (fMRI) or electroencephalography (EEG) [75]. Specifically, DCM assumes an underlying causal model with biophysically plausible properties and estimates its parameters via Bayesian inference [74].

Alternatively, we can assume, in a weaker sense, that information flow across or within brain regions is mapped into certain *meaningful* causal dependencies between neuron’s spike trains. By meaningful, we may understand that these dependencies map either anatomically direct or indirect neural interactions that are consistent with the processing of external stimuli or internally built actions (“information”) along a functional pathway (“flow”). Under this definition, we may include the biological interpretation employed in most of the studies reviewed in Section 3.5. Since GC-derived measures estimate causal dependencies, they can be used in this context but its application needs to be made with caution. Indeed, one of the main highlighted issues [76], [77], [67] is the fact that GC-derived measures only capture pairwise dependencies and hence, they conflate different sources of dependency when certain information is shared by more than two variables. This can be illustrated by a simple example given in [67]. Consider two sequences $X^{T}$ and $Y^{T}$, where $X^{T}$ is a sequence of independent and identically distributed Bernoulli variables with parameter p=1/2, and $Y^{T}$ is defined as follows:

(7) $$Y_{1}\sim \mathrm{Bern}(1/2)$$

(8) $$Y_{t}=X_{t-1}⊕Y_{t-1},\;2⩽t⩽T,$$

where ⊕ stands for the XOR operator between binary values 5. If we compute the DI between $X^{T}$ and $Y^{T}$ using the binary logarithm, we find that each term $I(Y_{t}\text{;}X^{t}|Y^{t-1})=1$ bit, for 1⩽t⩽T, and thus, the DI rate (4) equals 1bit, and as a non-zero quantity, it measures that $Y^{T}$ causally depends on $X^{T}$. However, a closer look at the model shows that the second argument of the conditional mutual information in (3), i.e, the truncated sequence $X^{t}$, cannot predict alone the variable $Y_{t}$ for any t⩾2. Hence, the estimated causal dependence is not uncovering a genuine information flow from $X^{T}$ to $Y^{T}$ because, in this example, it is the combination of the past of $Y^{T}$ and $X^{T}$ which contributes to the present of $Y^{T}$.

At the core of the above example, it lies the following theoretical fact: a straightforward application of the conditional mutual information fails in general to describe dependencies between random variables beyond pairwise interactions [77], [67] (e.g., in the above example there is a third-order dependence between $Y_{t},X^{t}$ and $Y^{t-1}$). This is indeed a critical problem in the field since a certain type of higher-than-two order interactions called synergistic have been found in several neuroscience studies [78], [79], [80]. To integrate these additional sources of interaction in the analysis, one can resort to the partial information decomposition (PID) framework proposed in [76]. Briefly, the PID decomposes the mutual information that a set of variables $A_{1},A_{2},\ldots ,A_{n}$ has about a variable B, i.e., $I(B\text{;}A_{1},A_{2},\ldots ,A_{n})$, into the information that the variables $A_{i}$ provide individually (unique information), redundantly (shared information) or only jointly (synergistic information) about B.

More recently, a more conceptual limitation of GC-derived measures is gaining attention in the literature. In our second information flow interpretation, we required that causal dependencies were part of a functional path that processed information content. In practice, if this information content is measurable we can make the requirement more specific and ask the estimated interactions to be statistically associated to an information message (an external stimulus, internal command, etc.), as it is considered in theoretical communication models [81]. In other words, causal dependencies need to be *about a message* [82]. Surprisingly enough, the effect of stimulus on estimated neural interactions has been to date largely neglected or uniquely considered as a source of covariation [21]. However, there is a growing consensus that the relationship between the stimulus (or any internal variable) and the estimated interactions is a necessary condition to support the information flow interpretation [64], [56], [83], [84], [82]. For instance, the use of GC-derived measures to analyze single-trial and time-varying neural interactions in monkeys performing perceptual decision-making tasks have showed the modulatory effect of stimulus information and internal percepts into inter-area interactions [64], [56].

## 5 New directions

In the following, we will overview two trends that have made recent progress in tackling some of the GC framework challenges discussed in Section 4. The first is motivated by current technology developments: it assesses whether we can benefit from large scale recordings and dimensionality reduction techniques to estimate functionally relevant neural interactions that are obscure to pairwise statistics. The second concerns the development of theoretical models and measures aimed to integrate the message statistics in order to improve the information flow interpretation.

### 5.1 Inferring multivariate interactions via dimensionality reduction

Over the last years, neural recording advances have made possible to record up to thousands of neurons simultaneously [85] keeping a pace that is growing at an exponential rate [5]. As a consequence, researchers have started to regard data analysis as a multi-dimensional problem with one of the key dimensions being the number of simultaneously recorded neurons. In this context, the classical notion of single-neuron activity has been replaced by that of population activity, which has been correlated with sensory stimuli, behavioral variables and between ensembles of simultaneous spike trains from different brain areas [86].

**Fig. 2:**
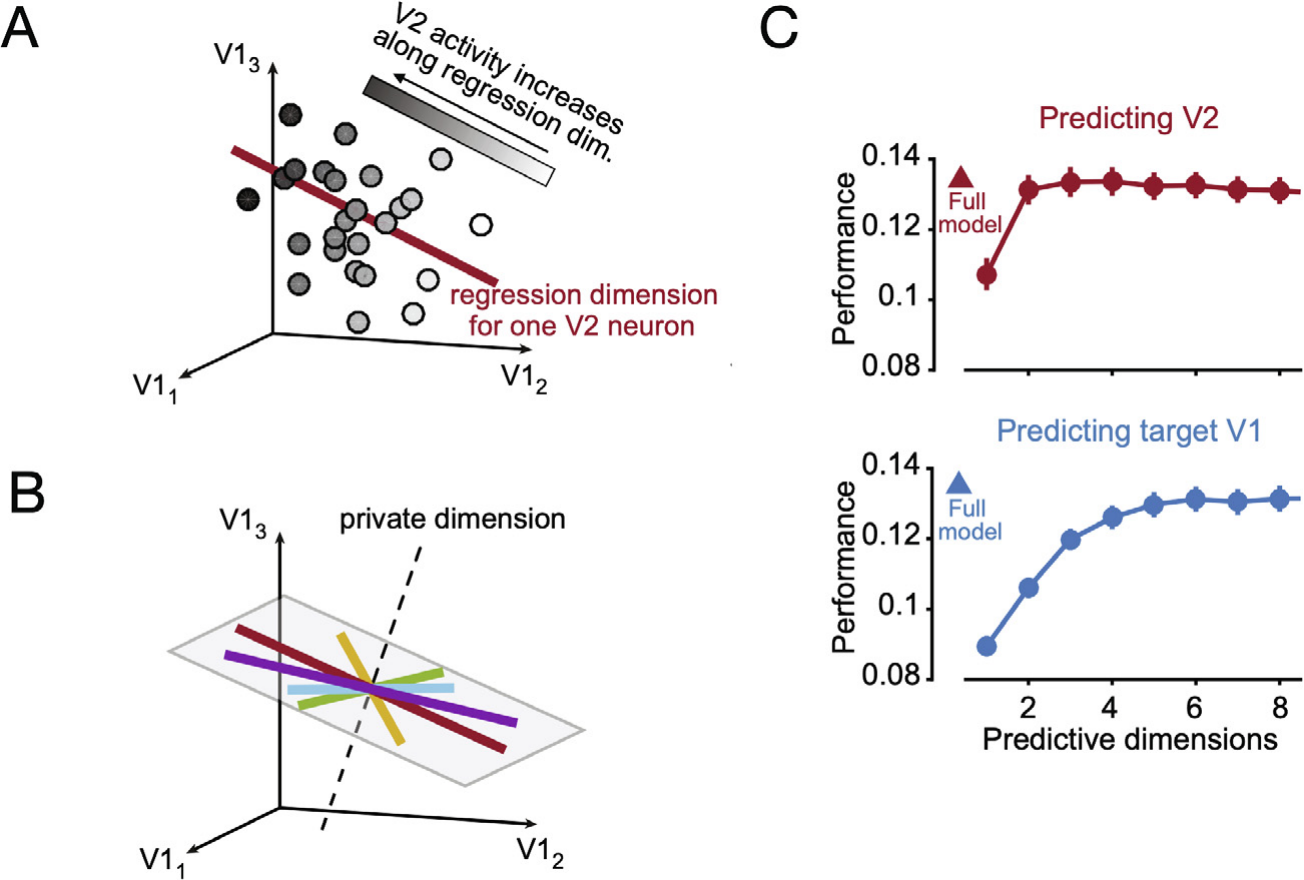
New directions: Inferring population interactions from multivariate datasets. Extracted from [96] (Fig.3 and Fig. 4) with permission. (A) Graphical depiction of linear regression between a population of V1 (primary visual brain area) neurons and one V2 (secondary visual brain area) neuron. Each circle represents the activity recorded simultaneously in V1 (three neurons) and V2 (one neuron) during an observation sample. The position of the circle represents the V1 population activity and its shading represents the activity of the V2 neuron. The activity of the V2 neuron increases along the regression dimension (red line). (B) Low-dimensional population interaction. The regression dimensions (shown as multiple-color straight lines) for different V2 neurons span a 2-dimensional subspace (the gray plane) of the V1 population space. Thus, two predictive dimensions are sufficient to capture the inter-area interactions between V1 and V2. All dimensions that are not predictive of V2, and therefore lie outside of this subspace, are called private dimensions. (C) Top: The number of predictive dimensions of V2 (red circles) in V1 needed to achieve full predictive performance (i.e., using all V1 neurons, red triangle) is two dimensions. Bottom: In contrast, the number of predictive dimensions of V1 (blue circles) in V1 needed to achieve full predictive performance (i.e., using all V1 neurons, blue triangle) is six dimensions. Error bars shows the standard error of the mean across different datasets. Adapted by permission from Elsevier Ltd. (For interpretation of the references to colour in this figure legend, the reader is referred to the web version of this article.)

A key aspect of this approach is the use of dimensionality reduction techniques to extract robust and interpretable information from multivariate recording sets [87], [88]. Examples of applied techniques are principal component analysis (PCA), factor analysis (FA) or tensor decomposition analysis [89], [90], [91], among others. Rather than spike trains, these techniques are typically applied over sequences of firing rates, obtained as the normalized number of spikes in a certain time window, which allows for multivariate Gaussian modelling. Using this framework, most studies have analyzed how distinct information features about stimuli [92], [93] or motor actions [94], [95] were encoded into lower-dimensional population activity subspaces, i.e. firing-rate subspaces that were of lower rank than the number of recorded neurons. In contrast, less work has been devoted to reformulate the study of spike-train dependencies at a population level and complement the above-reviewed approaches (GLM, GC). Yet, there are some interesting directions pointed in the recent literature [94], [96]. For instance, in the context of a motor task performed by macaque monkeys, Kaufman et al. investigated the communication mechanisms under which some information (muscle-related) lying in motor cortical areas flowed to the spinal cord and muscles, while some other (preparatory-related) largely stayed in the cortex [94]. Their analysis showed that the same population of neurons could project different sources of information (muscle or preparatory) into distinct activity subspaces, and these subspaces allowed to selectively route the appropriate information source (in this case, muscle-related) towards target regions such as the spinal cord and the muscles. Using similar methods, Semedo et al. more recently studied the structure of population interactions between the primary visual (V1) and the secondary visual (V2) brain areas in anesthesized macaque monkeys [96]. Their results concluded that V1 makes use of different population subspaces for intra-area and inter-areal interactions, respectively. In particular, they showed that the V1-V2 interaction subspace (named *communication subspace*) lying in V1 is of lower dimension and disjoint with respect to the V1 subspace capturing intra-area interactions (see Fig. 2). As a consequence, V2 population activity is related to a small subset of V1 population activity patterns, which differ from the most prevalent patterns shared by V1 neurons. These findings support the hypothesis introduced in [94] that neural population subspaces constitute a mechanism to route information across brain areas. Even though dimensionality reduction is a powerful ensemble of tools to deal with high-dimensional datasets, its current application to neuroscience has some limitations when it comes to inferring results in terms of information flow. To name a few, the lack of directionality or explicit stimulus variables in the models, and the special focus on PCA-related methods relying on Gaussian assumptions and variance maximization. In particular, to overcome the later issue, nonparametric generalizations of PCA such as projection pursuit [97] could be applied to ensembles of non-Gaussian firing rates for which functions other than the variance (e.g., skewness [98], entropy [99]) are optimized in order to unravel interesting lower-dimensional projections. Finally, dimensionality reduction techniques could be applied in non-linear models via the use of embedding methods [100].

### 5.2 Introducing the message variable in information flow models

The message, that is, the source random variable that needs to be transmitted over a network from an origin to a destination, is a key component in all theoretized communication models [81], [101], [102], Hence, when aiming to interpret spike trains dependencies as information flow, we may ask: What is the information source that these dependencies convey? To address this question, recent works [82], [84] have attempted to develop novel models and measures that infer the existence of information flow (or information transfer) by analyzing the interplay between recorded neural activity and the message variable (e.g., sensory stimulus) that is expected to flow.

**Fig. 3:**
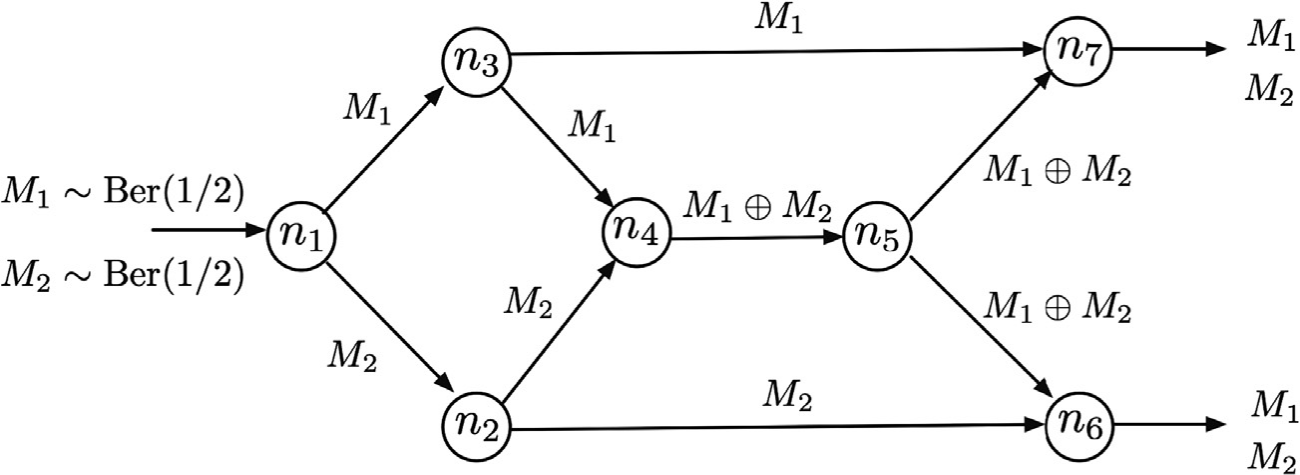
New directions: Information flow models with message variables and the need to address higher-than-two order dependencies. A depiction of the butterfly network introduced in [102] and used as an example of application in [82], in which higher-than-two dependencies arise among the observed variables. In this case, two binary messages $M_{1}$ and $M_{2}$ modeled as independent Bernoulli variables with parameter p=1/2 are transmitted from a source neuron ($n_{1}$) to two destination neurons ($n_{6}$ and $n_{7}$) travelling through intermediate nodes (illustrated with the corresponding message displayed on each travelled edge). Along the transmission, all neurons relay their incoming information except $n_{4}$, which performs the XOR operation of their incoming messages. For instance, in this network a third-order dependence may arise between the output activity of $n_{2}$ (neuron uniquely conveying $M_{2}$ information), $n_{3}$ (neuron uniquely conveying $M_{1}$) and $n_{4}$ (neuron uniquely conveying $M_{1}⊕M_{2}$). In contrast, it can be checked that all pairs among these 3 variables are marginally (pairwise) independent.

From a theoretical perspective and largely inspired by information theory models [81], [102], Venkatesh et al. have proposed a formal definition of information flow that explicitly includes the message as a model variable [82]. This definition is formulated in the framework of computational systems, which are defined as time-indexed directed graphs where node “transmissions” are modeled as random variables associated to their outgoing edges, where “computations” over each transmission are performed at each arriving node, and where there exists a subset of nodes (“input nodes”) whose transmissions depend on the message variable at time t=0. Then, information about the message flows on an edge as long as the mutual information between the corresponding edge random variable and the (discrete-valued) message conditioned on a set of additional edge variables has a positive value. The author’s definition is time-dependent since it assumes varying statistics over different observation time points and can be naturally extended to characterize information paths between pairs of nodes. Importantly, this approach specifically deals with the existence of the high-order dependencies reviewed in Section 4.2, which might arise between the observed edge variables and the message (see Fig. 3 for an exemplary network where this type of dependencies are present). Consistent with their definition, the authors provide an information flow inference method consisting of a set of conditional mutual information tests between the stimulus message and the recorded neural activity variables. Although the method might be in practice computationally costly and susceptible to common problems such as the effect of hidden variables, the overall proposal constitutes a valuable effort with theoretical and practical implications (see [82, Section VII]).

At a more practical level, Bim et al. tackled a similar problem and proposed a directed pairwise correlation measure that determines whether a causal dependence between two spike trains is about a certain stimulus feature [84]. In particular, their measure applies the notions of redundancy and uniqueness from PID theory [76], [77] as follows: it quantifies the information about the stimulus in the target spike train that is redundant with the information at the driver spike train and unique with respect to the information already available in the past activity of the target spike train. Consequently, this measure simultaneously addresses the presence of some high-order dependencies in the observed data and the required existence of information content during information transfer. However, because it strictly applies to pairs of spike trains, it cannot be a priori generalized to detect the variety of information flow mechanisms that might be present at a network level [102] (see also Fig. 3).

## 6 Summary and outlook

We have discussed the problem of modeling and inferring single-neuron and population interactions to detect neural information flows from the pioneering use of cross-correlations [8] to the most recent methods [72], [96], [82]. In particular, we have seen the evolution of model-based and model-free approaches to face technical estimation problems and allow meaningful biological interpretations. A special attention has been paid to the specificities and challenges of a widely established framework such as Granger causality. Finally, we have outlined new research lines that attempt to address some of the reviewed challenges.

As seen, this field has always been constrained by the technical difficulty of isolating the activity of multiple neurons from different brain areas simultaneously [103]. Critically, we are living an epoch of rapid technological advances in neural recordings [104] and the amount of available data requires improving the performance capacity and computing resources of current methods. In this paper, we have mainly referred to electrophysiological recordings (see Table 1 for a summary of applied studies with open-access data or software). However, it is due mentioning that a new generation of imaging recording methods relying on fluorescence molecule indicators [105] have been able to record the activity of more than 10,000 neurons simultaneously [106]. Consequently, these methods hugely increase the single-neuron recordings’ spatial resolution at the expense of reducing the temporal resolution [107] to detect spike trains 6. In conclusion, similar techniques like the ones reviewed here can be employed to analyze single-neuron interactions from imaging data as there are already examples in the literature [109], [110].

**Table 1:** Published applications to real spike-train data with open-access online data or software. V1: Primary visual area; V2: Secondary visual area. MT: Middle temporal area; LIP: Lateral intraparietal area; FEF: Frontal eye fields; A1: Primary auditory area; PFC: Prefontal cortex area; VPL: Ventral posterolateral nucleus of the thalamus; S1: Primary somatosensory area; PMd: Premotor cortex; M1: Primary motor area.

Method	Simultaneous dataset	Online data/software	Year
Cross-correlation [13]	V1 and V2 area neurons from anesthesized macaque monkeys during visual stimulation	doi.org/10.6080/K0B27SHN	2015
GLM [23]	In-vitro ganglion cells from macaque monkeys during visual stimulation	github.com/pillowlab/neuroGLM	2008
GLM [24]	MT and LIP area neurons from macaque monkeys performing a visual task	github.com/jcbyts/mtlipglm	2017
GLM [25]	Rat hippocampus during exploration of an open square field	github.com/NII-Kobayashi/GLMCC	2019
GLM [26]	LIP and FEF area neurons from macaque monkeys performing a visual task	doi.org/10.5061/dryad.gb5mkkwk7	2020
GC [72]	A1 and PFC area neurons from ferrets performing an auditory task	github.com/Arsha89/AGC_Analysis	2018
GC-DI [56]	VPL and S1 area neurons from macaque monkeys performing a somatosensory task	github.com/AdTau/DI-Inference	2019
Dim. reduction [94]	PMd and M1 area neurons from macaque monkeys performing a motor task	github.com/ripple-neuro	2014
Dim. reduction [96]	V1 and V2 area neurons from anesthesized macaque monkeys during visual stimulation	https://github.com/joao-semedo/communication-subspace	2019

Regardless of how neural data is recorded (e.g., electrophysiology or imaging techniques), there are different challenges that need to be tackled in the upcoming years. Below we outlook some of those from the conceptual and estimation angles, respectively. Conceptually, prior to following a model-based or model-free approach, it is critical to understand the limitations of the dataset at hand and appropriately define the notion of information flow that will be investigated in the study. Then, according to the defined notion, it is desirable to choose a proper method (e.g., GLM, DI, TE, PCA-based) and validate to a reasonable extent its assumptions on the data (e.g., trial independence, time series stationarity) to be able to make statistical inferences and interpretations [111].

There are still several statistical estimation issues that require further development such as the problem of non-stationarity data, the curse of dimensionality when aggregating multiple neurons, the observation noise, among others. However, recent developments such as combining data observation with model prior information (e.g., network sparsity, lower dimensionality activity) [72], [96], or simultaneously recording single neurons with surrounding aggregated neural activity [3], [112] have brought light to the above problems. An important aspect characterizing some of the methods reviewed in the paper is whether they use single or multiple trials to infer interactions associated to information flow. For instance, multiple trials are needed to evaluate dependencies between the information message and neural spike trains [82] because the former is usually variable only across trials. On the other hand, spike-train interactions should be validated at a single-trial level due to its possible variable statistics during repeated trials [56].

Due to the above mentioned limitations, spike-train inference methods are still far from providing a complete description of the spatial and temporal mechanisms by which multiple neurons communicate information between each other. Over the last two decades, we have experienced the rise and consolidation of GLM and GC approaches and we believe that we are about to witness a fruitful evolution of the topic in the next years thanks to novel theoretical [82] and practical insights [72], [96], [84]. This will eventually deepen our understanding on the inference of neural information flows, widen its application scope and provide a more unified approach to address biological questions by leveraging its connection to interactions estimated at larger recording scales [113], [114], [115], to computational models [86], [116], or to results obtained from other related paradigms such as neural population coding [117], [118], [119], [120] or network science [121].

## Declaration of competing interest

The author declares that he has no known competing financial interests or personal relationships that could have appeared to influence the work reported in this paper.

## Footnotes

1 For instance, time bin lengths of 1 ms may be selected so that a maximum of one spike falls per bin.

2 Note that a binary time series can be alternatively defined as a sequences of binary variables.

3 It is also known as Granger-Wiener causality to give credit to the earlier related work by N. Wiener [30].

4 The causality terminology of the GC framework is interpreted here in a fully statistical sense in contrast to the notion defined by J. Pearl' [32] that involves intervening the observed system.

5 The XOR operator satisfies 0⊕0=0,0⊕1=1,1⊕0=1,1⊕1=0.

6 Yet, there are recent improvements along this line [108].
